# The Important Role of Volatile Components From a Traditional Chinese Medicine Dayuan-Yin Against the COVID-19 Pandemic

**DOI:** 10.3389/fphar.2020.583651

**Published:** 2020-09-25

**Authors:** Xiao-rui Zhang, Ting-na Li, Yuan-yuan Ren, Yi-jia Zeng, Hong-yang Lv, Jin Wang, Qin-wan Huang

**Affiliations:** College of Pharmacy, Chengdu University of Traditional Chinese Medicine, Chengdu, China

**Keywords:** COVID-19, coronavirus, volatile components, aromatic Chinese herbs, Dayuan-Yin, traditional Chinese medicine

## Abstract

Aromatic Chinese herbs have been used to prevent plagues since ancient times. Traditional Chinese medicine has unique advantages in the prevention and treatment of epidemic diseases. According to the traditional Chinese medicine treatment plan in the National COVID-19 Diagnosis and Treatment Plan (Trial Seventh Edition) of the National Health Commission, Chinese patent medicines or prescriptions rich in aromatic Chinese herbs are selected for prevention and treatment during the period of medical observation, clinical treatment, and recovery of confirmed COVID-19 patients. Some local health committees or traditional Chinese medicine administrations recommend a variety of other ways of using traditional aromatic Chinese herbs to prevent and cure COVID-19. These involve external fumigation, use of moxibustion, and wearing of sachet. The efficacy of aromatic Chinese herbs plays a decisive role in the prevention and treatment of COVID-19. The unique properties, chemical composition, and mechanism of action of aromatic Chinese herbs are worthy of extensive and in-depth experimental and clinical research. The findings are expected to provide a reference for follow-up treatment of novel coronavirus and the development of corresponding drugs. In 2003, Dayuan-Yin produced excellent results in the treatment of the SARS virus. Individually, 112 confirmed cases were administered this drug between January and April 2003, and more than 93.7% of the patients showed noticeable mitigation of the symptoms, as well as recovery. Dayuan-Yin also was selected as one of the nationally recommended prescriptions for the COVID-19. Based on the national recommendation of Dayuan-Yin prescription, this review discusses the role of volatile components in the prevention and treatment of COVID-19, and speculates the possible mechanism of action, so as to provide a basis for the prevention and treatment of COVID-19.

## Introduction

When the new coronavirus infection broke out in Wuhan, China, in December 2019, WHO announced that it was PHEIC, which is named “COVID-19” (Wu et al., 2020). By mid-August 2020, more than 21,815,000 patients had been diagnosed with the disease worldwide, while 772,856 infected persons died. At present, the coronavirus has spread to 188 countries, with the US, Brazil, and India having a total of about 11,444,806 infected cases as of August 18, 2020 (Johns Hopkins University & Medicine, J.H.U, 2020). The situation is deteriorating every day, although the number of new cases in China has declined significantly since mid-March 2020. It is known that COVID-19 is harmful to different organs of the human body. Many governments have launched a joint prevention and control plan to prevent the spread of the COVID-19 pandemic.

Despite extensive and global scientific efforts, there is little drug has had a significant clinical effect on COVID-19 (Cao B. et al., 2020). Interestingly, Traditional Chinese medicine (TCM) plays an important role in the prevention, treatment, and rehabilitation of COVID-19 (Ren et al., 2020). According to the latest data from the State Administration of traditional Chinese medicine, Dayuan-Yin, a cocktail of aromatic Chinese herbs, has a significant therapeutic effect on COVID-19 (Ruan et al., 2020). In 2003, Dayuan-Yin produced excellent results in the treatment of the SARS virus. A total of 112 confirmed cases were individually administered Dayuan-Yin between January and April 2003, with more than 93.7% of the patients showing noticeable reduction in symptoms, as well as recovery (Li H. et al., 2020). As a result of this excellent therapeutic outcome, the TCM treatment plan in the National COVID-19 Diagnosis and Treatment Plan (Trial Seventh Edition) of the National Health Commission issued by the People’s Republic of China, has recommended Dayuan-Yin for normal COVID-19 patients (General Office of the National., Health Commission of the people’s Republic of China. et al., 2020). It has been used clinically in improving symptoms of lung condition for a long time, with the results showing that the prescription shortened the course of the disease by reducing the clinical symptoms and improving prognosis of patients. Thus, it is worthy of clinical application (Ruan et al., 2020; Wang W. et al., 2020). At present, the TCM has adopted Dayuan-Yin for the treatment of COVID-19, and it has achieved good curative effect (Wang B. et al., 2020; Li D. et al., 2020). The bioactive components of Dayuan-Yin remain unknown. This is probably due to the fact that the TCM decoction has nine herbal components derived from several prescriptions in a classic TCM fashion. The complex constituents of Dayuan-Yin make it hard to carry out a detailed study on its bioactive components in a short time.

**Figure 1:**
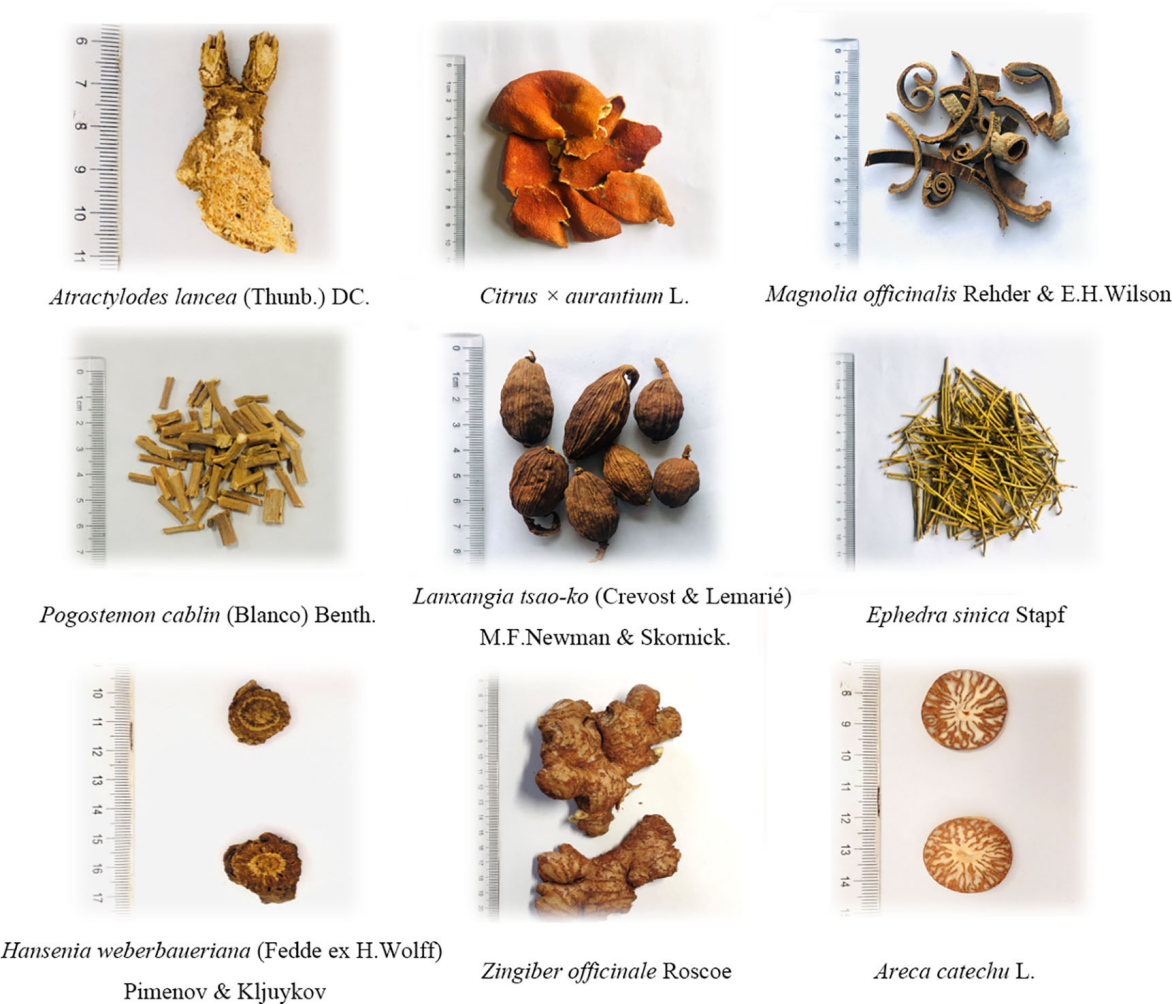
The processed raw materials of Dayuan-Yin in treating COVID-19.

These are Atractylodes lancea (Thunb.) DC., Citrus × aurantium L., Magnolia officinalis Rehder & E.H.Wilson, Pogostemon cablin (Blanco) Benth., Lanxangia tsao-ko (Crevost & Lemarié) M.F.Newman & Skornick., Ephedra sinica Stapf, Hansenia weberbaueriana (Fedde ex H.Wolff) Pimenov & Kljuykov, Zingiber officinale Roscoe, and Areca catechu L. in Dayuan-Yin. These plants are present in Dayuan-Yin in the ratio of 15: 10: 10: 10: 6: 6: 10: 10: 10. (General Office of the National., Health Commission of the people’s Republic of China. et al., 2020) (**Figure 1**). Eight of them are aromatic Chinese herbs.

Air pollution is a major environmental problem affecting global respiratory health (Guan et al., 2016). Moreover, aromatic Chinese herbs can be used for air disinfection (Sun and Liang, 2015). It is one of the reasons why aromatic Chinese medicine have been successfully used as key TCM for prevention of epidemics since ancient times (Luo et al., 2020). Volatile components are the main active components of aromatic Chinese herbs (Chen and Wang, 1994). It is supposed that the volatile components which are essential active ingredients in Dayuan-Yin may play a vital role in treating COVID-19 patients.

## Bioactive Volatile Components of Dayuan-Yin

Most of the volatile components of Dayuan-Yin have been elucidated, and their structures are well established (**Table 1**). However, there are no data on volatile components extracted from Areca catechu L. Based on clinical evidence of therapeutic results with Dayuan-Yin, we summarized its potential bioactive volatile components in the treatment of COVID-19. The biological benefits of Dayuan-Yin seem to involve anti-inflammatory, anti-viral, antibacterial, and immunomodulatory effects.

**Table 1:** Name of volatile oil in Dayuanyin prescription.

Volatile oils of herbs	Compounds	PubChem CID	References
*Pogostemon cablin* (Blanco) Benth. volatile oils	Patchoulol	10955174	(Lin et al., 2016; Tang et al., 2019)
	α-bulnesene	94275	
	α-guaiene	5317844	
	Seychellene	519743	
	α-patchoulene	521710	
	Carvacrol	10364	
	p-cymene	7463	
	γ-Terpinene	7461	
	β-guaiene	15560252	
	Eicosene	18936	
	Caryophyllene	5281515	
	Pogostone	54695756	
*Magnolia officinalis* Rehder & E.H.Wilson Cortex volatile oils	γ- eudesmol	6432005	(Tang et al., 2019)
	β- eudesmol	91457	
	α-pinene	6654	
	β-pinene	14896	
	Camphene	6616	
	Limonene	22311	
	Bornyl acetate	6448	
	Caryophyllene	5281515	
	Caryophllene Epoxide	14350	
	α-eudesmol	92762	
	Cryptomeridiol	165258	
	β-Caryophyllene	5281515	
	β-selinene	442393	
	Cardene	41114	
	β- cadinene	10657	
	2-Isopropenyl-4a,8-dimethyl-1,2,3,4,4a,5,6,7-octahydronaphthalene	605019	
	Eudesm-4-en-11-ol(8CI)	6432005	
volatile oils of *Pogostemon cablin* (Blanco) Benth. & *Magnolia officinalis* Rehder & E.H.Wilson Cortex	2-Cyclohexen-1-ol	13198	(Tang et al., 2019)
	1,7,7-trimethyl-bicyclo[2.2.1]hept-2-yl-ester	549130	
	Estragole	8815	
	2-Cyclohexen-1-ol,3-methyl-6-(1-methylethyl)-,cis;p-Menth-1-en-3-ol,cis-(8CI)	85567	
	Cadina-1,3,5,9-tetraene(8CI)	12302243	
	Cyclohexanemethanol,4-ethenyl-a,a,4-trimethyl-3-(1-methylethenyl)-,[1R-(1a,3a,4b)]-	92138	
	Hexamethylbenzene	6908	
*Atractylodes lancea* (Thunb.) DC. volatile oils	atractylon	3080635	(Yao et al., 2001; Guo et al., 2008; Chu et al., 2011)
	hinesol	10878761	
	α-phellandrene	7460	
	3- carene	26049	
	4-Biphenylcarbaldehyde	76689	
	Furanodiene	9601230	
	β- selinene	442393	
	β-eudesmol	91457	
	γ-elemene	6432312	
	α-elemol	6429032	
	Eudesma-4(14),11-diene	442393	
	aromadendrene	91354	
	β-sesquiphellandrene	12315492	
	patchoulene	91746471	
	atractylodin	5321047	
	α-pinene	6654	
	β-selinene	442393	
	Methyl palmitate	8181	
	Methyl linoleate	5284421	
	Methyl -9-octadecenoate	8202	
	Ethyl linoleate	5282184	
	4-hydroxyphthalic acid	11881	
	β-selinene	442393	
*Citrus × aurantium* L. eticulatae Pericarpium volatile oils	limonene	22311	(Jing et al., 2015; Castro et al., 2018; Vieira et al., 2018; Pan et al., 2020)
	sesquiterpene	6473767	
	α-pinene	6654	
	β-pinene	14896	
	sabinene	18818	
	γ-Terpinene	7461	
	β-myrcene	31253	
*Lanxangia tsao-ko* (Crevost & Lemarié) M.F.Newman & Skornick. volatile oils	1,8-Cineole	2758	(Xiong and Hua, 2012)
	4- Propylbenzaldehyde	120047	
	Geraniol	637566	
	Geranial	638011	
	α-Terpineol	17100	
	α-Phellandrene	7460	
	β-Pinene	14896	
	α-Pinene	6654	
	α-phellandrene	7460	
	2-Propenal,3-methyl-3-phenyl	5372857	
	neral	643779	
	Cineole	2758	
	γ-terpineol	11467	
	β-citral	643779	
	α-citral	638011	
	2-decenal	147309	
	6-methyl-1,2,3,5,8,8a·hexahydronaphthalene	562093	
	2-oxaadamantane	520365	
	2-phenyl-2-butenal	6429333	
	geraniol acetate	1549026	
	*trans*-2-undecen-1-ol	5365004	
	2-dodecenal	5283361	
	*trans*-nerolidol	5284507	
	mint furanone	91753282	
	hedycaryol	6432240	
	cyclohexanol,2-methylene-3-(1-methylethenyl)-,acetate,cis-	22213002	
	Citral	638011	
*Hansenia weberbaueriana* (Fedde ex H.Wolff) Pimenov & Kljuykov volatile oils	α-Pinene	6654	(Wu et al., 2008)
	β-Pinene	14896	
	D-Limonene	440917	
	α-Bisabolol	1549992	
	β-Ocimene	18756	
	β-Bisabolene	10104370	
	γ-Terpinene	7461	
	β-Thujene	520384	
	α-Terpinolene	11463	
	Terpinen-4-ol	11230	
	1-Bornyl acetate	93009	
	α-Copaene	70678558	
	Trans-β-farnesene	5281517	
	Apiol	10659	
	Guaiol	227829	
	Benzyl benzoate	2345	
	5-Allyl-2,3-(methylendioxy)anisole	4276	
	β-Cedarene	11106485	
	Spathulenol	97032059	
	9,12-Octadecadienoic acid(Z,Z)-,ethyl ester	5282184	
	Palmitoleic acid	445638	
	α- phellandrene	7460	
	3-carene	26049	
	1-Methyl-2-(1-Methylethyl)Benzene	10703	
	3,7-dimethyl-1,3,6-octatriene	5281553	
	1-lsopropyl-2-methoxy-4-methyl-benzene	14104	
	Z-3-decen-1-ylacetate	5363204	
	Agarospirol	21675005	
	Bicyclo[3.1.1]heptane, 6,6-dimethyl-2-methylene-, (1S)-	24848167	
	Benzene, 1-methyl-3-(1-methylethyl)-	10812	
	Azulene, 1,2,3,3a,4,5,6,7-octahydro- 1,4-dimethyl-7-(1-methylethenyl)-,	90805	
	o-Cymene	10703	
	β-Phellandrene	11142	
Ginger essential oil	camphene	6616	(El-Ghorab et al., 2010)
	p-cineole	2758	
	α-terpineol	17100	
	zingiberene	92776	
	pentadecanoic acid	13849	
*Ephedra sinica* Stapf volatile oil	2,3,5,6-tetramethylpyrazine	14296	(Kun et al., 2000; Xue et al., 2020)
	linalool	6549	
	4-methoxystyrene	12507	
	α-terpineol	17100	
	myrtenol	10582	
	geraniol	637566	
	(4-Isopropyl-2-cyclohexen-1-yl)methanol, trans-	22215197	
	phytol	5280435	

**Table 2:** The mechanism of action of volatile components in Dayuanyin prescription.

Bioactivities	Volatile oils of herbs	Mechanisms	References
Anti-inflammatory activity	*Pogostemon cablin* (Blanco) Benth. volatile oils	Patchoulene: cyclin E↓, cyclin B↓, CDK1↓; the subsequent S-phase arrest, IFN-gamma↓, IL-10↓	(Su et al., 2015)
		*Pogostemon cablin* (Blanco) Benth. volatile oils: TNF-α↓, IL-6↓, IL-10↑, NP-SH↑	(Chen et al., 2015)
		Patchoulene: regulates on the balance between Nrf2 and NF-κB p65 signaling pathways	(Yang et al., 2018)
		Patchoulene: vitro neutrophil fMLP chemotaxis↓, phagocytic activity↑; ear edema↑, myeloperoxidase (MPO) activity↑	(Silva-Filho et al., 2016)
		Patchoulene: NF-κB↓, Nrf2↑, miR-146a expression↑	(Chen et al., 2017)
		Patchoulene: NF-κB↓, p38 MAPK phosphorylation↓	(Li et al., 2014)
		Patchoulene: regulates on the balance between Keap1-Nrf2 and NF-κB signaling pathways	(Sun et al., 2016)
	*Magnolia officinalis* Rehder & E.H.Wilson Cortex volatile oils	*Magnolia officinalis* Rehder & E.H.Wilson Cortex volatile oils: PGE2/TNF-α↓, IL-1β↓	(Cao et al., 2015)
	*Atractylodes lancea* (Thunb.) DC. volatile oils	Atractylone: caspase-1/NF-κB/MAPKs activations↓, reduce IL-1↓, IL-4↓, IL-5↓, IL-6↓, IL-13↓, COX-2↓, intercellular protein-2 expression↓	(Kim et al., 2016)
		Atractylone: RPMCs degranulation intracellular Ca2+ level ([Ca2+])↓, tryptase↓, histamine↓ p56lck tyrosine kinase activity↓; histidine decarboxylase activity and expression↓, tryptase and histamine releases↓in PMACI-induced HMC-1 cells; morphological alteration and flamentous actin formation in stem cell factor-stimulated RPMCs animal model	(Han et al., 2016)
		Atractylone: NLRP3 inflammasome↓, TLR4 activation↓	(Tang et al., 2018)
		Hinesol: H^+^, K^+^ -ATPase activity↓	(Kanako et al., 2000)
	*Citrus × aurantium* L. Pericarpium volatile oils	Limonene: TNF-α↓, neutrophils chemotaxis↓, leukocytes chemotaxis↓	(Vieira et al., 2018)
		Limonene: iNOS↓, COX↓, PGE2↓; TNF-α↓, IL-1β↓, and IL-6↓;	(Yoon et al., 2010)
	*Lanxangia tsao-ko* (Crevost & Lemarié) M.F.Newman & Skornick. volatile oils	*Citrus × aurantium* L. Pericarpium volatile oils: Nitric oxide↓, iNOS↓, COX-2↓	(Dang et al., 2020)
		1,8-cineole: IL-4↓, IL-5↓, IL-10↓, MCP-1↓, IL-1 beta↓, IL-6↓, TNF-alpha↓, IFN-gamma↓, NF-kB p65↓, ICAM-1↓; VCAM-1↓ in lung tissues of mice infected with influenza A virus.	(Li et al., 2016)
		1,8-cineole: mucin-filled goblet cells↓, MUC2↓, MUC19↓, NF-kappa B-activity↓	(Sudhoff et al., 2015)
		1,8-cineole: IL-10↑, TNF-α↓, IL-1β↓, NF-κB’s subunit p65↓ and TLR4↓	(Zhao et al., 2014)
		1.8-cineole: LTB4↓ and PGE2↓in human blood monocytes ex vivo in the treatment of bronchial asthma.	(Juergens et al., 1998b)
		1.8-cineole: TNFαC, IL-1β↓, leukotriene B4↓, thromboxane B2↓ in human blood monocytes *in vitro*	(Juergens et al., 1998b)
	*Hansenia weberbaueriana* (Fedde ex H.Wolff) Pimenov & Kljuykov volatile oils	*Hansenia weberbaueriana* (Fedde ex H.Wolff) Pimenov & Kljuykov volatile oils: NO↓ in RAW 264.7 cells.	(Bi et al., 2018)
		α-pinene: MAPKs↓, NF-κB↓ in mouse peritoneal macrophages	(Kim et al., 2015)
		α-pinene: LPS-induced nuclear translocation of NF-κB↓ in TPS-1 cells by κBα expression↑ in a dose-dependent manner	(Zhou et al., 2004)
	Ginger volatile oil	PLS-induced IL-8 secretion↓, RANTES↓ in human bronchial epithelial cells (BEAS-2B)	(Podlogar and Verspohl, 2012)
Antiviral activity	*Pogostemon cablin* (Blanco) Benth. volatile oils	anti-Coxsackie virus (IC50 0.081 mg/ml, TI 1.25), anti-adenovirus (IC50 0.084 mg/ml, TI 1.20), anti-influenza A virus (IC50 0.088 mg/ml, TI 1.15), and anti-respiratory syncytial virus (IC50 0.092 mg/ml, TI 1.10)	(Wei et al., 2012)
	*Atractylodes lancea* (Thunb.) DC. volatile oils	Regulate the TLR7 signaling pathway.	(Cheng et al., 2016)
	*Lanxangia tsao-ko* (Crevost & Lemarié) M.F.Newman & Skornick. volatile oils	1,8-cineole: IRF3 antiviral activity↑, proinflammatory NF-κ B signalling↓	(Müller et al., 2016)
Anti-oxidative activity	*Magnolia officinalis* Rehder & E.H.Wilson Cortex volatile oils	Scavenging DPPH free radicals, providing hydrogen atoms, scavenging superoxide free radicals	(Guo, 2012)
	*Atractylodes lancea* (Thunb.) DC. volatile oils	Scavenge DPPH- radical activity with an IC50 of 288.7 μg/mL, lipid peroxidation↓, and effects on T-AOC in the serum and organ tissues of mice	(He et al., 2020)
	*Citrus × aurantium* L. Pericarpium volatile oils	Citrus reticulata peel oil prevented LDL lipid peroxidation because oxLDL are absorbed by the macrophages’ scavenger molecules, forming foam cells	(Yoon et al., 2010; Castro et al., 2020)
		Certain monoterpenes and essential oils: LDL oxidation↓	(Barter, 2005)
		Limonene: protect the lens epithelial cells from oxidative stress through antioxidant and anti-apoptotic pathways.	(Naderi. et al., 2004)
		Limonene: be able to attenuate the oxidative stress impairment on *in vitro* and *in vivo* models	(Ahmad and Beg, 2013)
	*Lanxangia tsao-ko* (Crevost & Lemarié) M.F.Newman & Skornick. volatile oils	Weak in scavenge DPPH- radical activity, TBA, and FRAP	(Yang et al., 2010)
		1,8-cineole: reactive oxygen species↓, superoxide dismutase↓, catalase↓, malondialdehyde↓	(Kennedy-Feitosa et al., 2016)
Anti-bacterial activity	*Pogostemon cablin* (Blanco) Benth. volatile oils	Inhibit Candida albicans (MIC 0.9 ml/L), Crytococus neoformans (MIC 0.15 ml/L), Sporothrix schemckii (MIC 0.6 ml/L), Microporum lanosum (MIC0.7 ml/L), M.gypseum (MIC 0.6 ml/L), Aspergillus flavus,AS3.3950 (>1.0 ml/L), A.niger,AS3.3928) (MIC>1.0 ml/L, Mucor globosum,AS3.963 (MIC 1.0 ml/L), Chaetomium globosum,AS3.963 (MIC 0.45 ml/L), Rhizopus nigricans, AS3.31 (MIC 0.8 ml/L), Scopulariopsis brevicaulix (MIC0.5 ml/L), Escherichia coli, 8099 (MIC>1.0 ml/L), Bacillus subtilis, ATCC 9379 (MIC 0.7 ml/L), Staphylococcus albus, AS1.184 (MIC 0.8 ml/L),Micrococcus tetrgenus (MIC 0.8 ml/L),Staphylococcus aureus (MIC 0.7 ml/L),methicillin-resistant Staphylococcus aureus, Staphylococcus epidermidis (MIC 0125 mg/ml), Shigella sonnei standard strain (MIC 0125 mg/ml), Hemolytic Streptococcus A, Pseudomonas aeruginosa, Bacillus subtilis, yeast, penicillium.	(Zhou et al., 2014; Lin et al., 2016; Wang et al., 2018)
	*Magnolia officinalis* Rehder & E.H.Wilson Cortex volatile oils	Inhibit Staphylococcus aureus, Candida albicans, methicillin-resistant Staphylococcus aureus, Staphylococcus epidermidis, Enterococcus faecalis, Shigella sonnei, Escherichia coli, Listeria monocytogenes, Salmonella, Bacillus cereus, Pseudomonas aeruginosa.	(Guo, 2012; Tang et al., 2019)
	*Atractylodes lancea* (Thunb.) DC. volatile oils	Inhibit Gram-positive and Gram-negative bacteria due to disruption of the cell membrane.	(He et al., 2020)
		β-eudesmol has two-way regulation of gastrointestinal motility, which may be anticholinergic or directly acting on gastrointestinal smooth muscle.	(Wang et al., 2002)
		Hinesol: H^+^, K^+^-ATPase activity↓	(Kanako et al., 2000)
	*Citrus × aurantium* L. Pericarpium volatile oils	Inhibit the phytopathogenic fungus *Sclerotinia sclerotiorum*.	(Dias et al., 2020)
		
	*Lanxangia tsao-ko* (Crevost & Lemarié) M.F.Newman & Skornick. volatile oils	Protect the mice from Staphylococcus aureus or Escherichia coli infection.	(Dai et al., 2016)
		1,8-cineole: inhibits *S. aureus, Escherichia coli, Moraxella catarrhalis*.	(Schürmann et al., 2019)
	Ginger volatile oil	Inhibit Pseudomonas aeroginosa bacteria, S. typhimurium and S. flexneri, Gram-negative bacteria, slightly.	(Mesomo et al., 2013)
Other aspects	*Lanxangia tsao-ko* (Crevost & Lemarié) M.F.Newman & Skornick. extract	Epicatechin has anti-inflammatory properties, quercetin has the strongest neuroprotective effect of PC-12 cells induced by H_2_O_2_, and DPPH radical-scavenging activity.	(Zhang et al., 2014)
	*Hansenia weberbaueriana* (Fedde ex H.Wolff) Pimenov & Kljuykov extract	Inactivate the influenza virus A/FM/1/47 directly and reduce the titer.	(Guo et al., 2005)
		Falcarindiol inhibited DC maturation by blocking the canonical pathway of nuclear factor-kappaB and phosphorylated p38.	(Mitsui et al., 2010)
		Falcarindiol inhibit Pseudomonas aeruginosa by repressing virulence-related genes, including the T3SS; quorum sensing synthase genes lasIR and rhlIR; lasB; motility-related genes fliC and fliG; and phenazine synthesis genes phzA1 and phzA2.	(Zhang et al., 2020)

### Anti-Viral Effect

In autopsy studies and animal models, COVID-19 manifests mainly as acute viral pneumonia leading to respiratory failure (Chan et al., 2020; Yao et al., 2020). Antiviral drugs have been used to treat common cold, fever and influenza viruses by destroying the viral surface structure and inhibiting its entry (Hsieh et al., 2012), suggesting that antiviral drugs can be used for COVID-19. Unfortunately, no specific antiviral treatment has been recommended for COVID-19 treatment because of insufficient evidence from randomized trials (Hung et al., 2020). It has been shown that many re-purposed drugs have effects against close relatives of SARS-COV-2, such as β-coronavirus, in vitro. Furthermore, lopinavir and many interferons, especially interferon beta, have moderate effects against SARS-COV in vitro and can be used in combination with ribavirin (Chen et al., 2004; Chan et al., 2013). Administration of antiviral drugs soon after symptoms appear reduces the release of virus in respiratory secretions of patients with COVID-19, thereby decreasing their infectivity to others. Targeted preventive treatment for contacts reduces their risk of infection (Welliver et al., 2001; Oriol and Bonaventura, 2020).

Patchouli oil is extracted from *Pogostemon cablin* (Blanco) Benth. Some studies in vitro have shown that patchouli oil exerted anti-viral effects against Coxsackie virus (IC_50_ = 0.081 mg/ml, TI 1.25), adenovirus(IC_50_ = 0.084 mg/ml, TI 1.20), influenza A virus (IC_50_ = 0.088 mg/ml, TI 1.15), and respiratory syncytial virus(IC_50_ = 0.092 mg/ml, TI 1.10) (Wei et al., 2012). Evaluation of the antiviral properties of six chemical compositions of *Atractylodes lancea* (Thunb.) D DPPH.C. revealed that atractylodin produced the most significant effect at doses of 10–40 mg/kg for five days, and attenuated IAV−induced pulmonary injury via regulation of the TLR7 signaling pathway (Cheng et al., 2016). Moreover, 1,8-cineole, the major constituent of the essential oil of *Lanxangia tsao-ko* (Crevost & Lemarié) M.F.Newman & Skornick., is commonly applied for treating inflammatory diseases of the respiratory tract caused by viruses since it potentiates the antiviral effect of IRF3, in addition to its inhibitory effect on proinflammatory NF-κB signaling (Müller et al., 2016).

### Anti-Inflammatory Effect

The levels of proinflammatory factors i.e. IL-2, IL-7, IL-10, GCSF, IP10, MCP1, mip1a, and TNF – α in the plasma of critically-ill patients were higher than those in plasma of patients who were not in intensive care, suggesting that “cytokine storm” is closely related to the severity of COVID-19 (Huang et al., 2020). *Cytokine storm* is a very prominent pathophysiological feature of COVID-19 infection (Lin et al., 2020). Extensive endothelial barrier disruption and uncontrolled *cytokine storm* promote uncontrolled inflammatory response which is the basis of the core mechanism underlying acute respiratory distress syndrome (ARDS) (Huang et al., 2017), although this phenotype varies among individuals. Experimental models of acute lung injury (ALI) and human genome-wide association studies of ARDS indicate that *cytokine storm* plays an essential role in the pathophysiology of ARDS ( Biondi et al., 1986; Huang et al., 2017). Moreover, the most common and severe complication of COVID-19 is ARDS (Huang et al., 2017). Therefore, an understanding of the *cytokine storm* that aggravates ARDS in COVID-19 may lead to early and effective intervention in critically-ill COVID-19 patients. It seems necessary to directly inhibit inflammatory response in the lungs because *cytokine storm* can be alleviated with inflammatory therapy (Cao P. et al., 2020). Previous studies have shown the benefits of anti-inflammatory drugs in lung disease: they slow down impairment of lung function, reverse the inflammatory parameters nearly back to normal values, and improve patients’ survival (Konstan et al., 2011; Lubamba et al., 2011). Ibuprofen, a popular anti-inflammatory drug, is recommended for airway inflammation in cystic fibrotic lung disease (Flume et al., 2007). Studies have shown that long-term prediagnostic use of nonaspirin NSAIDs (e.g., ibuprofen) is associated with a significant reduction in lung cancer survival (Brasky et al., 2012). In addition, barictinib, fedratinib, and ruxolitinib are active and selective JAK inhibitors which have been approved for rheumatoid arthritis and myelofibrosis. All three drugs are effective anti-inflammatory agents, and as JAK-STAT signaling inhibitors, they may be effective against the consequences of elevated levels of cytokines (including interferon-γ) usually observed in patients with COVID-19 (Stebbing et al., 2020). The UK is currently conducting a randomized evaluation of COVID-19 treatment (recovery) trial, based on the announcement on June 16, 2020, that dexamethasone had been shown to significantly improve the prognosis of COVID-19 patients receiving respiratory support (Recovery, Randomised Evaluation of COVid-19 therapy trial, 2020). Dexamethasone is a glucocorticoid which can be used as a synthetic form of the natural hormone cortisol (Cain and Cidlowski, 2017). It has the same anti-inflammatory effect as cortisol. It inhibits the release of inflammatory chemokines by immune cells, thereby improving the prognosis of patients by reducing the severity of ARDS (Lester et al., 2020). In European patients, low-dose dexamethasone reduces mortality by 33% in critically patients requiring invasive ventilation (Lim et al., 2020). However, the implementation of appropriate dexamethasone use in low-and-middle-income countries has been a challenge. For example, corticosteroids may cause sepsis in some prevalent parasitic infections in Africa (Nutman, 2020). Therefore, the use of dexamethasone in African patients who have not been diagnosed with COVID-19 may lead to unexpected consequences (Brotherton et al., 2020). During the treatment of COVID-19, Dayuan-Yin also reduces the severity of ARDS by inhibiting the release of inflammatory chemokines from immune cells. As a classic prescription in ancient China, Dayuan-Yin can play a safe and effective role in the treatment of respiratory infections in a more adverse environment. Thus, it can avoid such problems in a large extent.

Anti-inflammatory property is widespread in various sources of volatile components. Several data have found that 1,8-cineole significantly improved lung function and health conditions, and reduced dyspnea in patients with asthma, acute bronchus, and chronic obstructive pulmonary disease (COPD). Moreover, it significantly reduced the frequency of cough in patients with acute bronchitis, and alleviated frequent exacerbations in patients with COPD and frequent exacerbations, notably (Worth et al., 2009; Worth and Dethlefsen, 2012; Fischer and Dethlefsen, 2013; Vogelmeier et al., 2018). In a mouse model of LPS-induced acute pulmonary inflammation, 1,8-cineole upregulated IL-10 in lung tissues, and decreased the expressions of TNF-α, IL-1β, NF-κB’s subunit p65 and TLR4 (Zhao et al., 2014). Moreover, 1,8-cineole was shown to inhibit LTB4 and PGE2 (pathways of AA-metabolism in human blood monocytes) in bronchial asthma in vitro (Juergens et al., 1998b). In addition, 1,8-cineole decreased levels of TNFα, IL-1β, leukotriene B4, and thromboxane B2 in human blood monocytes in vitro (Juergens et al., 1998a).

Pogostone, a bioactive component extracted from *Pogostemon cablin* (Blanco) Benth., reduced the total population of T cells under ConA stimulation by blocking T cell proliferation via down-regulation of cyclin E, cyclin B, and CDK1. Subsequent S-phase arrest inhibited the production of IFN-gamma and IL-10 (Su et al., 2015). Simultaneously, pogostone pretreatment mitigated ethanol-induced gastric ulcer in rats by downregulation of IL-6 and TNF-alpha, and upregulation of IL-10 and non-protein-sulfhydryl (NP-SH) groups in the gastric mucosa (Chen et al., 2015). In lung disease, pogostone exerted potent protective effects against lipopolysaccharide-induced acute lung injury in mice by decreasing TNF-α-induced cell injury in A549 cells through modulation of the balance between Nrf2 and NF-κB-p65 signaling pathways (Yang et al., 2018). Pogostone significantly inhibited the protein and mRNA expressions of proinflammatory mediators such as TNF-α, IL-6, IL-1β, NO, and PGE2. Pogostone also significantly reduced LPS-induced mortality in mice, suppressed the production of proinflammatory mediators in serum. And it attenuated liver and lung injury via downregulation of the mRNA expressions of inflammatory mediators in multiple organs due to inhibition of activation of NF-κB and phosphorylation of p38 MAPK (Li et al., 2014). Pre-treatment with pogostone markedly mitigated LPS-induced acute lung injury in mice, improved survival, attenuated histological alterations in the lungs, reduced MPO and MDA levels, decreased the wet/dry weight ratio of lungs, and down-regulated proinflammatory mediators, such as TNF-a, IL-1 beta and IL-6. Furthermore, pretreatment with pogostone enhanced the Nrf2-dependent genes NQO-1, GCLC, and HO-1, but suppressed the NF-kappa B regulated genes TNF-alpha, IL-1 beta, and IL-6. The mechanism involved in the protective effect of pogostone was correlated with its regulation of the balance between Keap1-Nrf2 and NF-kappa B signaling pathways (Sun et al., 2016). Moreover, volatile oils from *Pogostemon cablin* contain a bioactive component named β-patchoulene which has been shown to significantly decrease mortality and lung wet/dry weight ratio of mice, and mitigate pathological changes in lungs, when compared to model group. It suppressed LPS-induced activation of NF-kappa B, and markedly upregulated Nrf2 and miR-146a (Chen et al., 2017).

### Anti-Oxidative Properties

Oxidative stress and inflammation form a positive feedback cycle (Mittal et al., 2014). In lung disease, excessive inflammation and oxidative stress lead to adverse outcomes. For instance, patients with COPD are usually affected by other diseases (Rabe and Watz, 2017). Several mechanisms in lung inflammation and oxidative stress destroy DNA and lead to an imbalance between tissue repair and cell proliferation, which seems to promote the link between COPD and lung cancer (Wilson et al., 2008; Houghton, 2013; Durham and Adcock, 2015). Under normal conditions, the production and elimination of ROS maintain a crucial balance between oxidation and antioxidation (Cao et al., 2019). In such a balance, the signal pathways are regulated, and cell proliferation can be guaranteed. When inflammatory factors destroy this balance, oxidative stress enhances the maturation of proinflammatory factors, leading to oxidative damage to cells and multisystem diseases (Sies, 2015; Kruk et al., 2019). Antioxidant drugs have been used in lung diseases. For example, antioxidants have been recommended for reduction of mortality or prevention of organ damage in animal models of acute lung injury induced by lipophilic acids (Hsu et al., 2006; Zhu et al., 2020). Vitamin C has also been shown to reduce the incidence of pneumonia in several controlled trials for human subjects (Hemila, 2017).

The essential oil of *Magnolia officinalis* Rehder & E.H. Wilson exerts antioxidant effect by scavenging 1,1-diphenyl-2-picrylhydrazyl (DPPH) radical and superoxide anion radical. The essential oil contains β–eucalyptol with a hydroxyl group which can provide hydrogen atom for scavenging DPPH radical. Moreover, with increase in volatile oil concentration, the antioxidant capacity gradually increased (Guo, 2012). The essential oil from *Atractylodes lancea* (Thunb.) DC. showed a strong antioxidant effect in vitro and indicated by DPPH-radical scavenging property, with an IC_50_ of 288.7 μg/ml. Moreover, it inhibited lipid peroxidation, and affected total antioxidant capacity (T-AOC) in the serum and organ tissues of mice (He et al., 2020). In short-term cigarette smoke (CS)-induced acute lung inflammation, 1,8-cineole decreased oxidative stress involving reactive oxygen species, by increasing superoxide dismutase and catalase, while reducing levels of malondialdehyde, inflammation, and the NF-kappa B p65 subunit (Kennedy-Feitosa et al., 2016).

### Antibacterial Properties

The major components of *Pogostemon cablin* (Blanco) Benth. are carvacrol (47.5%) and p-cymene (15.2%). It completely inhibits the growth of *E. coli* at a level of 0.05% (Lin et al., 2016). The essential oil of *Atractylodes lancea* (Thunb.) DC. exhibited antibacterial effects against Gram-positive and Gram-negative bacteria due to the cell membrane (He et al., 2020). In chronic rhinosinusitis, 1,8-cineole suppressed the growth of *S. aureus*, *Escherichia coli*, *Moraxella catarrhalis* due to downregulation of significant and critical players in biofilm generation (Agra, Sara, and σ^B^) (Schürmann et al., 2019). On the other hand, the major constituent of the essential oil of *Atractylodes lancea* (Thunb.) DC. is β-eudesmol. In terms of intestinal flora, β-eudesmol has two-way regulation for gastrointestinal motility: anticholinergic pathway and direct effect on gastrointestinal smooth muscle.

Antibacterial effect is an essential pharmacological property of volatile compounds (Houdkova et al., 2017; Riad et al., 2020). Secondary bacterial co-infection is common in patients with COVID-19 infection, and it leads to adverse prognosis (Macintyre et al., 2018). At present, many antibiotics have been used in the treatment of COVID-19. For example, Shufeng Jiedu Capsule (SFJD) prevents acute upper respiratory tract infection and positively affects fever, cough, and headache. Studies have shown that SFJD significantly reduced the levels of serum PGE2, IL-1 β, and TNF-α in rats with acute pharyngitis (Qian, 2019). Being a popular antiviral and antibacterial drug, SFJD is one of the drugs for COVID-19 treatment in China (Pan X. et al., 2020). In addition, Shuanghuanglian (SHL) is a popular anti-bacterial drug. It has various pharmacological potential such as antibacterial, antiviral, and immune-enhancing properties which can be exploited in the treatment of acute upper respiratory tract infection (Zhang et al., 2013). Preliminary studies in vitro showed that SHL oral liquid inhibited SARS-COV-2. Indeed, SHL has been used to carry out clinical research on COVID-19 in Shanghai Public Health Clinical Center and Tongji Hospital Affiliated to Huazhong University of Science and Technology (Pan X. et al., 2020).

The antibacterial effects of volatile components of Dayuan-Yin are not limited to upper respiratory tract infections: these volatile components also regulate intestinal flora, treat gastric ulcers, and improve gastrointestinal symptoms. Human gut microbes are the “second genome” of the human body (Backhed et al., 2005; Gill et al., 2006; Cani and Delzenne, 2007). The composition of intestinal flora is closely related to human health status, and it plays an essential role in maintaining physiological balance (Schuijt et al., 2016). It has been confirmed that intestinal flora reduces ventilator-associated pneumonia and enteritis by enhancing the function of primary alveolar macrophages (Bradley et al., 2019). Patients with COVID-19 showed intestinal microbial malnutrition and decreased microbial flora levels of some probiotics such as *Lactobacillus* and *Bifidobacterium*. The latest version of novel coronavirus pneumonia diagnosis and treatment plan released by the People’s Republic of China National Health Council suggests that intestinal microbiota should be used in severe and critical cases to maintain intestinal micro ecological balance (General Office of the National., Health Commission of the people’s Republic of China. et al., 2020).

## Effects of Non-Volatile Components

Apart from the biological effects of the volatile components mentioned above, other non-volatile components of Dayuan-Yin also have abundant pharmacological properties.

Epicatechin, one of the chemical components of *Lanxangia tsao-ko* (Crevost & Lemarié) M.F.Newman & Skornick., exhibited excellent anti-inflammatory properties in LPS-stimulated macrophage RAW 264.7 cells. Quercetin, one of the chemical components of *Lanxangia tsao-ko* (Crevost & Lemarié) M.F. Newman & Skornick., produced the most potent neuroprotective effect on PC-12 cells induced via H_2_O_2_ and DPPH radical-scavenging properties (Zhang T. T. et al., 2014). It was shown that SFE-CO_2_ extract of *Hansenia weberbaueriana* (Fedde ex H.Wolff) Pimenov & Kljuykov significantly prolonged average survival time of mice with influenza virus pneumonia, directly killed the influenza virus, and reduced the hemagglutination titer. *Hansenia weberbaueriana* (Fedde ex H.Wolff) Pimenov & Kljuykov induced immunosuppressive effects in vitro. Falcarindiol, the main bioactive compound in *Hansenia weberbaueriana* (Fedde ex H.Wolff) Pimenov & Kljuykov, inhibited DC maturation by blocking the canonical pathway of nuclear factor-kappaB and phosphorylated p38 (Mitsui et al., 2010). Falcarindiol inhibited the growth of *Pseudomonas aeruginosa* by repressing virulence-related genes, including the T3SS; quorum sensing synthase genes lasIR and rhlIR; lasB; motility-related genes fliC and fliG; and phenazine synthesis genes phzA1 and phzA2 (Zhang et al., 2020).

It is obvious that Dayuan-Yin exerts extensive biological properties such as antiviral, anti-inflammatory, antioxidative, and antibacterial effects. It can be inferred that Dayuan-Yin may play an essential role in preventing COVID-19 pandemic.

## Summary and Future Prospects

At present, there are no conventional drugs that can cure COVID-19 (Cao B. et al., 2020). However, according to data collected by the National Health Commission of the people’s Republic of China, clinical practice in Chinese hospitals have reported that traditional Chinese medicine has a definite therapeutic effect in the early stages of COVID-19 infection (Liu C.-X. et al., 2020). As a significant part of medical practice, Chinese medicine has been used to treat human diseases for more than 5,000 years (Li and Kan, 2017). In recent decades, volatile compounds extracted from medicinal plants have attracted more and more attention due to their important biological effects such as antiviral, anti-inflammatory, and antibacterial properties. Besides, they are non-toxic and have few side effects, making them suitable for use as drugs. This review discussed the potential role of traditional Chinese medicine in terms of volatile components. The anti-inflammatory, antiviral, antibacterial and immunomodulatory effects of these volatiles seem to play the most critical roles in treating patients infected with COVID-19. However, there are still lack of clinical trials on Dayuan-Yin. These need to be done in future.

In China, the situation of COVID-19 pandemic prevention and control has improved. The national pandemic situation has been controlled. However, with the resumption of factory work, re-opening of shopping malls, and resumption of transportation, the cross-flow of personnel has increased significantly, and the probability of close contact between people has increased tremendously too. In particular, with likelihood of increase in imported cases from abroad, the epidemic prevention and control should not be relaxed. It is essential to improve the ability of the human body to withstand infection. In addition to frequent washing of hands, wearing masks, social distancing and other measures, the “Chinese medicine sachet” can be used as an essential means of prevention. This stems from a very important theory of traditional Chinese medicine, namely “treating pre-disease”. This idea in traditional Chinese medicine originated in the Yin and Shang Dynasties, took shape in Zhouyi, and formed in Huangdi Neijing (Xu et al., 2016). Chinese doctors in the past dynasties attached great importance to the prevention and treatment of diseases. They emphasized the prevention of diseases first, especially infectious diseases (Lian et al., 2020). Wearing *Chinese medicine sachet* is another special treatment of “treating pre-disease” (Chen et al., 2020). *Chinese medicine sachet* has been used to prevent disease since ancient times. In this method, aromatic Chinese medicine is put into a unique bag and worn on the chest to prevent respiratory diseases. This is known as “Xiangpei therapy” (Zhang Q. et al., 2014). From the perspective of modern medicine, the medicinal fragrance (i.e., volatile oil components) of Chinese medicine sachet stimulates the nasal mucosa, promotes the secretion of immunoglobulins, and kills all kinds of viruses at the same time, thereby playing multiple roles in regulating immune function, and exerting antibacterial and anti-viral effects (Lvy and Bai, 2017). Interestingly, early intervention with aromatic Chinese medicine blocks the course of diseases and relieves symptoms in clinical practice through oral administration, external fumigation, and moxibustion (Lun and Chen, 1987; Chen et al., 2013). Aromatic Chinese medicine dispels exterior pathogenic factors, regulates *qi*, activates blood circulation, *breaks blood stasis*, and *disperses nodules*. The application of aromatic Chinese medicine embodies the theory of “internal disease and external treatment” of traditional Chinese medicine (Hu et al., 2010). Since the outbreak of COVID-19, fumigation has been used for air disinfection to prevent the spread of the virus. In the clinical treatment period, the application of moxibustion plays the role of anti-inflammatory agent, regulates immune function, and prevents deterioration of the patients (Zhang, 2012; Liu K. et al., 2020). Some local health committees or Chinese medicine administration bureaus are actively involved in promoting aromatic traditional Chinese medicine as an anti-epidemic, as well as the use of fumigation or *Chinese medicine sachet* to prevent and control COVID-19 (Chen et al., 2020).

There is no doubt that the pharmacological effects of volatile components of traditional Chinese medicine are beneficial in the global fight against COVID-19. However, each TCM prescription has multiple goals and links in the treatment of diseases, making it difficult to clearly and thoroughly explain its mechanism in a short period. More research should be carried out on volatile components of traditional Chinese medicine to elucidate the associated regulatory mechanism, evaluate possible side effects, and conduct standard clinical trials. The insights provided in this review may help ease the COVID-19 pandemic worldwide.

## Author Contributions

Q-wH and JW are the corresponding authors on the study. X-rZ and T-nL are first authors and responsible for collecting materials and writing the paper. Y-yR, Y-jZ, and H-yL helped in organizing the information and edited the article pictures. All authors contributed to the article and approved the submitted version.

## Funding

This work is financially supported by the Xinglin Scholar Talent Promotion Plan of Chengdu University of Traditional Chinese Medicine (QNXZ2018023, XSGG2019008).

## Conflict of Interest

The authors declare that the research was conducted in the absence of any commercial or financial relationships that could be construed as a potential conflict of interest.

## Abbreviations

ALI, acute lung injury; CDK1, cyclin-dependent kinases 1; CDK1, cyclin-dependent kinases 1; COPD, chronic obstructive pulmonary disease; COX, cyclooxygenase; COX-2, cyclooxygenase; DPPH, 1,1-diphenyl-2-picrylhydrazyl; fMLP, N-formyl methionyl leucyl phenylalanine; FRAP, ferric reducing antioxidant power; FRAP, ferric reducing antioxidant power; IAV, influenza A virus; IC50, 50%inhibiting concentration; ICAM, intercellular adhesion molecule; ICAM-1, intercellular adhesion molecule; IFN, Interferon; IFN-gamma, Interferon-gamma; IL, interleukin; IL-10, interleukin-10; IL-1β, interleukin 1 beta; IL-6, interleukin-6; iNOS, inducible nitric oxide synthase; iNOS, Nitric oxide synthase; IRF3, Interferon regulatory Factor 3; LDL, low-density lipoprotein; LTB4, Leukotriene B4; MAPKs, mitogen activated protein kinases; MCP, monocyte chemotactic protein; MIC, minimum inhibitory concentration; MUC, mucoprotein; NF-κB, nuclear factor-kappa B; NLRP3, Nucleotide-binding domain-(NOD-) like receptor protein 3; NO, nitric oxide; NP-SH, non-protein-sulthydry; Nrf2, nuclear factor erythroid 2-related factor 2; NSAIDs, nonsteroidal anti-inflammatory drugs; oxLDL, lipid peroxidation because oxidized LDLs; PGE2, prostaglandin E2; PGE2, prostaglandin E2; PHEIC, a public health emergency of international concern; RANTES, regulated upon activation normal T-cell expressed and secreted; RPMCs, Rat peritoneal mast cells; ROS, reactive oxygen species; SARS, Severe Acute Respiratory Syndrome; SFJD, Shufengjiedu Capsule; SHL, Shuanghuanglian; T3SS, type III secretion system; TBA, thiobarbituric acid; TCM, traditional Chinese medicine; TI, therapeutic index; TLR4, toll like receptor 4; TLR4, toll like receptor 4; TLR7, toll like receptor 7; TNF-alpha, tumor necrosis factor-alpha; TNF-α, tumor necrosis factor alpha; VCAM-1, vascular cell adhesion molecule; WHO, Worldwide Health Organization.
